# Effect of Rubber Particle Size and Content on the Mechanical Properties of Rubber–Clay Mixtures Solidified by EICP

**DOI:** 10.3390/ma18153429

**Published:** 2025-07-22

**Authors:** Qiang Ma, Meng Li, Chen Zeng, Hang Shu, Lei Xi, Yue Tao, Xuesong Lu

**Affiliations:** 1 School of Architectural Engineering, Huanggang Normal University, Huanggang 438000, China; 2 Key Laboratory of Intelligent Health Perception and Ecological Restoration of Rivers and Lakes, Ministry of Education, Hubei University of Technology, Wuhan 430068, China; 3 School of Civil Engineering and Architecture, Wuhan Polytechnic University, Wuhan 430023, China

**Keywords:** soil solidification, enzyme-induced carbonate precipitation, rubber–clay mixtures, mechanical properties

## Abstract

Using the enzyme-induced carbonate precipitation (EICP) technique to solidify rubber and clay mixtures as lightweight backfill is a feasible way to reduce waste tire impacts and boost rubber recycling in geotech engineering. In this study, a comprehensive laboratory investigation, including triaxial compression, oedometer, permeability, and nuclear magnetic resonance (NMR) tests, was conducted on EICP-reinforced rubber particle solidified clay (hereafter referred to as EICP-RC solidified clay) to evaluate the effects of rubber particle content and size on the mechanical behavior of the improved soil under various solidification conditions and to elucidate the solidification mechanism. The results show that although rubber particles inhibit EICP, they significantly enhance the mechanical properties of the samples. The addition of 5% rubber particles (rubber A) increased cohesion by 11% and the internal friction angle by 18% compared to EICP-treated clay without rubber. Additionally, incorporating smaller-sized tire particles facilitated pore filling, resulting in lower compression and swelling indices and reduced permeability coefficients, making these materials suitable for use behind retaining walls and in embankment construction.

## 1. Introduction

Two controversial issues related to cohesive soil are its inherent low shear strength and high compressive deformation. These challenges could be addressed through the implementation of well-established chemical stabilization techniques. The use of chemical stabilizers, such as cement or lime, enhances stiffness and induces brittle behavior in treated soils [1]. Incorporating reinforcing materials (such as polypropylene fibers and lignin fibers) presents an effective and feasible approach for tackling the issues related to high stiffness and brittleness [2]. Despite the satisfactory results of chemical additives in the mechanical strengthening of cohesive soil, the toxic effects on the environment, ecosystems, and human beings should not be overlooked, especially the excessive carbon emissions from the reaction of cement and lime. Consequently, it is imperative to explore new reinforcing materials that offer environmental benefits while providing strength, resilience, and enhanced friction resistance. The search for environmentally friendly soil improvement methods has been driven by the themes of “low carbon” and “carbon neutrality”.

In the past few years, a substantial amount of research has been carried out on numerous environmentally friendly technologies, among which biocementation through urea hydrolysis is considered an emerging method for soil improvement with a reduced CO_2_ footprint, less detrimental to the environment and human health compared to traditional chemical treatments [3,4]. For example, Microbial-Induced Carbonate Precipitation (MICP) and Enzyme-Induced Carbonate Precipitation (EICP) have been extensively studied for enhancing soil properties [5]. MICP relies on urease produced by microorganisms. Conversely, EICP directly employs the extracted urease to catalyze the requisite reactions, thereby eliminating the need for associated bacteria [6]. Furthermore, the size of the extracted urease, which is typically 0.012 μm, is notably smaller than that of the enzyme produced by microorganisms, usually ranging from 0.3 to 0.5 μm. This size-related advantage confers upon it adaptability to a broader spectrum of soils and simplifies the application procedures. Another crucial point to be noted is that competition might exist between the indigenous soil microorganisms and the bacteria introduced through the MICP method [7]. Therefore, the EICP method offers significant advantages over the MICP method in terms of environmental friendliness and efficiency. It is suitable for a wider range of soils and features a more straightforward application process [8,9]. Numerous recent investigations have demonstrated that the EICP method exhibits efficacy in enhancing the strength and stiffness of sand [10,11,12,13]. Nevertheless, the majority of these studies have centered on sandy soils, with only a handful exploring the treatment of fine-grained soils. In cohesive soils, engineering issues such as low strength, high compressibility [14], significant settlement [15], and the proneness to swelling and shrinking in response to seasonal variations [16,17] are prevalent. Therefore, conducting research on the application of EICP in clayey soil is of utmost importance.

Simultaneously, the global disposal of used tires presents significant environmental and health challenges. Annually, a substantial number of tires are discarded, with only a small percentage undergoing recycling. The majority are disposed of in landfills without proper treatment [18], thereby occupying considerable amounts of land. Some waste tires are incinerated without effective reuse, resulting in the emission of gases such as carbon monoxide, sulfur dioxide, and trace amounts of carcinogenic compounds, including polycyclic aromatic hydrocarbons. It is evident that improper stacking or burning of waste tires can lead to significant environmental issues and ultimately pose a serious threat to human health [19]. The appropriate management and disposal of used and waste rubber tires continue to be major resource and environmental challenges in the 21st century.

To alleviate the negative influences of scrap rubber tires and adhere to a sustainable development strategy, there has been an escalating emphasis on the recycling of these solid wastes. Over the past three decades, numerous researchers have employed waste rubber for soil enhancement due to its favorable geotechnical properties and environmental benefits. Considering the characteristics of waste rubber, such as high durability, low density, and suitable strength, these wastes are also utilized in geotechnical engineering by blending with clays to enhance their engineering properties. It has been observed that incorporating fragments of waste rubber tires into cohesive soil reduces its mass while increasing its shear strength [20,21]. This indicates that mixing waste tire fragments with clay can serve as an effective filler material. Owing to the fact that the density of rubber particles is lower than that of clay particles, as the proportion of rubber particles in the mixture rises, the overall density of the mixture diminishes. The soil incorporated with recycled tire waste is capable of being utilized as a lightweight filling material for slopes, pavement sub-bases, and retaining walls [22]. Rubber tire debris exerts a notable impact on the one-dimensional compressive properties of cemented clay. This is achieved by influencing the vertical effective stress and modifying the compression and expansion coefficients within the composite material [23]. Cui et al. [24] conducted research on the application of rubber particles to ameliorate the brittle behavior of bio-cemented calcareous sands. The findings demonstrated that rubber particles were efficacious in enhancing the brittle behavior of bio-cemented calcareous sands, as well as in delaying and reducing dilation. Chen et al. [25] discovered that the incorporation of rubber particles has the ability to enhance the shear strength, cohesion, and internal friction angle of EICP-treated loess. When it comes to the improvement of loess, it is advisable to select rubber particles with a particle size ranging from 1 to 2 mm and a rubber particle content of approximately 10%. It must be emphasized that prior research has predominantly concentrated on the improvement of special soils. As a result, there is a deficiency in the comprehensive geotechnical property analysis of common clays.

To more effectively investigate and utilize the practical applications of this composite material, this study aims to elucidate the synergistic effects of rubber particle size and content on the mechanical and hydraulic properties of EICP-reinforced clay-rubber composites (EICP-RC). Through a comprehensive laboratory investigation encompassing triaxial compression, oedometer, permeability, and nuclear magnetic resonance (NMR) tests, we systematically quantify the influence of rubber characteristics on: (i) shear strength parameters (cohesion and internal friction angle), (ii) compressibility and swelling indices, (iii) permeability coefficients, and (iv) microstructural pore evolution. The composites developed provide a viable solution for small road projects, meeting the growing demand for new construction materials while reducing the environmental burden through the recycling of used tires, promoting sustainability and environmental friendliness.

## 2. Materials and Methods

### 2.1. Materials

#### 2.1.1. Soil

**Figure 1:**
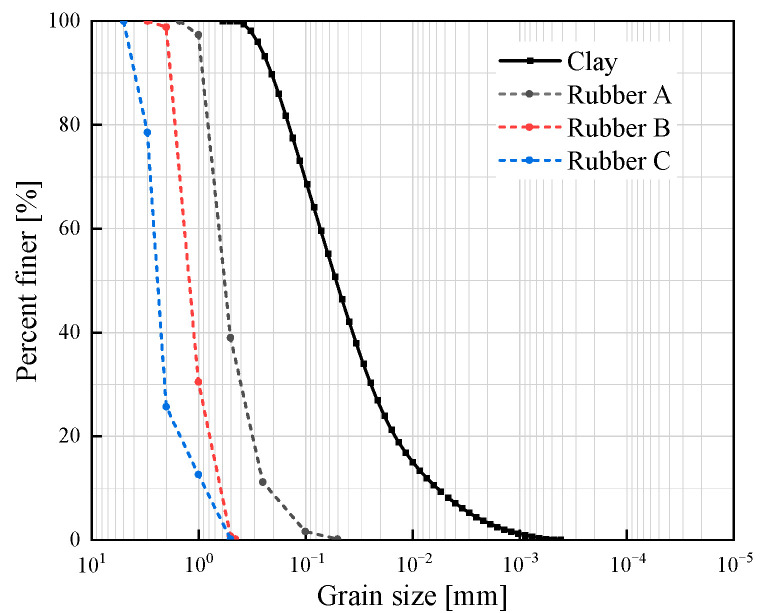
Particle size distribution of the clay and crumb rubber.

**Figure 2:**
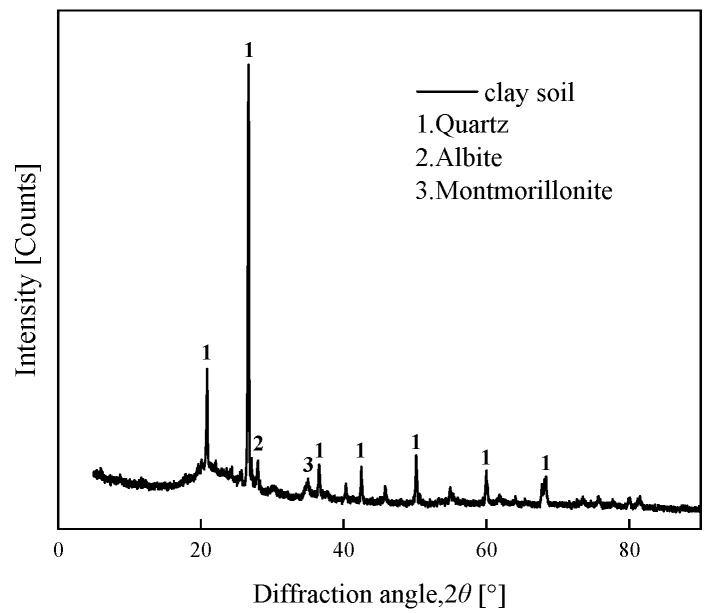
XRD diagram of the clay.

The soil sample, classified as Quaternary alluvial clay from the Middle Pleistocene epoch (Q_2_), was collected from a depth of approximately 10 m at an excavation site in Hankou District, Wuhan City, Hubei Province (30°35′ N, 114°17′ E). This region lies within the Jianghan Plain, a major Quaternary depositional basin of the Yangtze River system. The clay exhibited a characteristic yellowish-brown coloration, consistent with the iron oxide-rich, fluvio-lacustrine sediments typical of this geological formation. The soil exhibited a yellowish-brown coloration. After undergoing a drying process, the fundamental physical properties of the soil were assessed and are summarized in Table 1. The particle size distribution curve of the soil employed for testing is illustrated in Figure 1. X-ray fluorescence spectrometry (XRF) was utilized to ascertain the soil composition, and the obtained results are shown in Table 2. The X-ray fluorescence spectrometer is purchased from Riken of Japan (Rigaku, Akishima, Japan). The instrument model is ZSX Primus III+ 50KV 60mA. More than 50 percent of the soil was composed of SiO_2_, followed by Al_2_O_3_, which accounted for 18.75 percent of the mass. The XRD analyses indicated that the soil was predominantly composed of quartz, sodium feldspar, and montmorillonite clay minerals, and the results are presented in Figure 2.

#### 2.1.2. Rubber Particle

**Figure 3:**
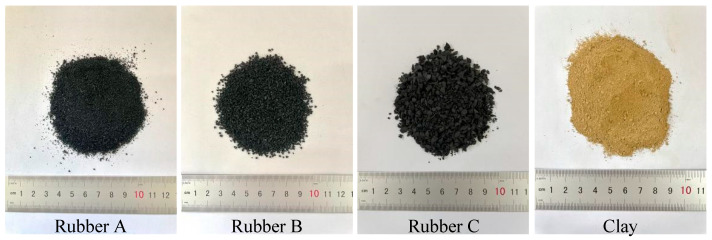
Different sizes of crumb rubber and clay used in this study.

In this study, the rubber particles originated from Huayi Rubber Co., Ltd. (Chengdu, China) and were prepared by crushing waste tires, and their size distribution is shown in Figure 1. Three distinct gradations of waste rubber particles, generated from crushed waste tires, were investigated. As depicted in Figure 3, the particle sizes of Rubber A (*d*_50_ = 0.59 mm), Rubber B (*d*_50_ = 1.28 mm), and Rubber C (*d*_50_ = 2.46 mm) are close to the particle sizes of fine sand (0.075–0.425 mm), medium sand (0.425–2 mm), and coarse sand (2–4.75 mm), respectively. The coefficient of inhomogeneity (*C_u_*) and the coefficient of curvature (*C_c_*) for Rubber A were determined to be *C_u_* = 2.95 and *C_c_* = 1.12. In contrast, for Rubber B and Rubber C, the coefficients were *C_u_* = 2.20 and *C_u_* = 2.94, respectively, with *C_c_* values of 1.05 and 1.81, respectively. Based on the United Soil Classification System (USCS) standards, the gradations of the three rubber materials under investigation can all be categorized as being equivalent to poorly graded sand (SP). In addition, the specific gravities of rubber A, rubber B, and rubber C were 1.09, 1.11, and 1.13, respectively. The rubber particles used in this study were irregularly shaped.

#### 2.1.3. EICP Solution

The EICP solution was formulated through the combination of equal-volume portions of the EICP cementing solution and the urease solution, followed by the addition of skimmed milk powder to provide nucleation sites [26]. Analytical grade urea and calcium acetate were employed in this study. Urease extracted from jack beans with an activity level of 150 U/mg was typically stored within a temperature range of 2–8 °C. The EICP cementing solution was formulated by combining urea and calcium acetate in a 1:1 molar ratio. Considering the inhibitory effects of high-concentration salt solutions on urease activity, as well as their influence on the physical properties of cohesive soil [27], a lower concentration of the EICP cementing solution (0.5 M urea, 0.5 M calcium acetate, 0.684 g/L urease, and 4 g/L skimmed milk powder) was used in the aim of enhancing the efficacy of EICP consolidation. The activity of the urease solution was determined by means of the electrical conductivity (EC) method [28], which corresponds to 4.54 urea min^−1^ of urease activity.

### 2.2. Specimen Preparation

**Figure 4:**
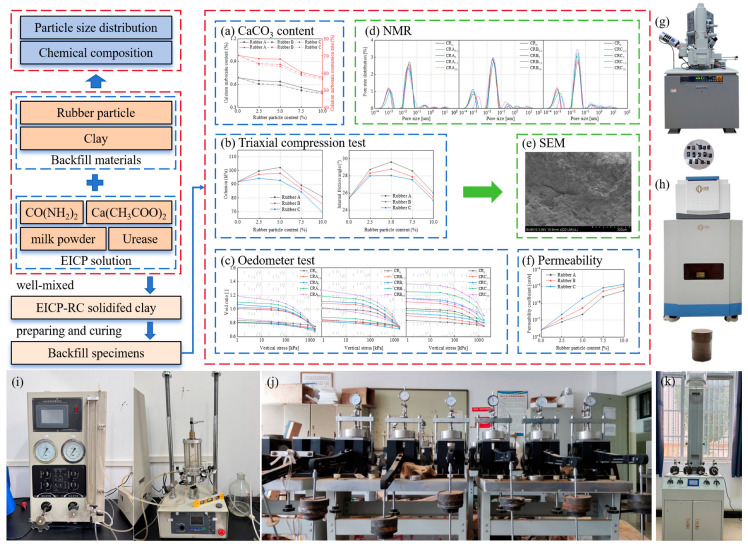
Test procedures, results and test apparatus: (**a**) the determination result of calcium carbonate content; (**b**) test results of the triaxial compres-sion test; (**c**) test results of the oedometer test; (**d**) test results of the nuclear magnetic resonance test; (**e**) microstructure of the sample; (**f**) test results of the permeability test; (**g**) the instrument for mi-crostructure testing; (**h**) the instrument for nuclear magnetic resonance test; (**i**) the instrument for triaxial compression testing; (**j**) the instrument for oedometer test; (**k**) the instrument for permeability test.

In this study, specimens with rubber particle content ranging up to 15% were initially prepared. It was observed that samples containing more than 10% rubber particles disintegrated upon removal from the mold, and the highly elastic rubber particles readily returned to their original state after the load was removed [20]. Based on the test results, the rubber particle content in the mixture was maintained at 10%. The clay retrieved from the site was dried in an oven and sieved through a 2 mm sieve. Subsequently, rubber particles accounting for 2.5%, 5%, 7.5%, and 10% (relative to the dry weight of the soil) were added to the dry clay, and the mixture was thoroughly blended. The modified Proctor compaction test was employed to ascertain the maximum dry density and optimum water content of the diverse combinations of clay and rubber particles. The results are presented in Table 3. In accordance with the optimum moisture content of the mixture, the EICP solution was incorporated into it and thoroughly blended. Subsequently, the resultant mixture was compacted into the mold by means of a hydraulic jack and left to stand for a duration of one minute. Once the molding process was completed, the samples were extracted from the mold and sealed with plastic film. Subsequently, the prepared samples were positioned within the curing box maintained at a constant temperature of 20 °C and a relative humidity of 95% for a period of 14 days. After 14 days, the enzyme has been largely inactivated, and curing is considered complete. To better understand the results, a mixture code was used. EICP-RC solidified clay are denoted by three capital letters, with the third letter indicating the type of rubber particle and the subscripted value representing the percentage of rubber particles in the mixture. For example, the mixture “CRA_2.5_” signifies a clay mixture containing 2.5% of rubber A. Where “CR_0_” refers to clay mixtures without rubber particles treated only with EICP. The test procedure and test apparatus are shown in Figure 4.

### 2.3. Test Methods

The triaxial compressive and oedometer tests were prioritized to address the study’s focus on shear strength and compressibility, which are critical for backfill materials. Permeability tests were included to evaluate drainage characteristics, ensuring compatibility with applications requiring controlled water flow (such as embankments). Combining oedometer tests with NMR allowed a holistic understanding of macro-mechanical behavior and microstructural mechanisms. This dual-scale approach is well-established in geotechnical research to bridge performance metrics with material science insights.

#### 2.3.1. Determination of Calcium Carbonate Content

The acid wash method was selected to quantify calcium carbonate (CaCO_3_) content due to its reliability in distinguishing carbonate minerals from organic and silicate phases. This method is widely adopted in bio-cementation studies to evaluate the efficiency of EICP reactions [29]. Following the triaxial testing, around 20 g of the sample was collected. Subsequently, it was rinsed multiple times with deionized water to eliminate soluble salts. Thereafter, the samples were placed in an oven for drying until a constant mass, denoted as *m_a_* was attained. Afterwards, the dried samples were subjected to repeated washing with 0.5 M dilute hydrochloric acid using filter paper. This process continued until the reaction no longer generated bubbles. Finally, the sample was washed several times with deionized water. The residue was captured on filter paper and then placed in an oven for drying until a constant mass, denoted as *m_b_* was achieved. The disparity in the weight of the samples prior to and subsequent to the acid-wash procedure was regarded as the mass of the generated calcium carbonate. The calcium carbonate content can be computed using Equation (1). In the present study, we put forward the calcium carbonate conversion rate as a metric to evaluate the reaction extent of EICP. The CaCO_3_ conversion rate is calculated by Equation (2):

(1) $$CCC=(1-\frac{m_{b}}{m_{a}})\times 100\%$$

where *CCC* is the CaCO_3_ content, *m_a_* and *m_b_* are the masses of the samples before and after acid washing, respectively.

(2) $$\mathrm{ConversionRate}(CR)=M_{\mathrm{Actualprecipitation}}/M_{\mathrm{Theoretical}\;\mathrm{precipitation}}$$

where *CR* is the CaCO_3_ conversion rate, M_Actual precipitation_ and M_Theoretical precipitation_ are the actual and theoretical precipitation mass of CaCO_3_, respectively.

#### 2.3.2. Triaxial Compressive Test

Consolidated drained (CD) triaxial tests were conducted using the TSZ-2 strain-controlled apparatus (Nanjing Geotechnical Instrument & Equipment Co., Ltd., Nanjing, China) to evaluate the shear strength and stress–strain behavior of EICP-RC solidified clay under varying confining pressures (50, 100, and 150 kPa). The CD test regime was chosen to simulate long-term field conditions where drainage is permitted, aligning with the intended applications of lightweight backfill materials in retaining walls and embankments. A strain rate of 0.4 mm/min was selected to prevent pore pressure buildup and ensure quasi-static loading, consistent with ASTM D7181 standards [30,31]. Prior to the testing process, each specimen was made completely saturated through the subsequent method: (1) The specimens were vacuum saturated for 24 h prior to testing; (2) hydraulically saturated for 30 min after installation on the apparatus; and (3) counterpressure saturated until at least 95 per cent saturation was achieved. The specimens were then consolidated under a specific enclosure pressure. After consolidation was complete, axial compression was performed at the specified rate until an axial strain of 20% was reached.

#### 2.3.3. Oedometer Test

To elucidate the deformation characteristics associated with the settlement resilience of mixed soil, oedometer tests were conducted on specimens using loading-unloading stress paths in a WG lever consolidation instrument (Nanjing Geotechnical Instrument & Equipment Co., Ltd., Nanjing, China), following ASTM D2435 [32]. Prior to testing, the samples were saturated via vacuum back pressure. To keep the specimens in a saturated condition throughout the test, they were submerged in distilled water while the loading process was taking place. Vertical stresses were incrementally applied at levels of 12.5, 25, 50, 100, 200, 400, 800, and 1600 kPa, followed by unloading in the reverse order. This method provides critical insights into the long-term deformation behavior of rubber–clay mixtures, which is essential for predicting settlement in practical applications.

#### 2.3.4. Permeability Test

Under saturated conditions, the performance of soils is significantly influenced by their drainage characteristics. Therefore, permeability tests were performed on the specimens by means of the falling head method. This method ensures accurate determination of flow rates under saturated conditions, which is vital for evaluating the material’s suitability as a hydraulic barrier or drainage layer. Prior to testing, the specimens underwent vacuum backpressure saturation and were subsequently placed in a permeation test apparatus. The water flow through the specimen was measured by monitoring the decrease in the water level inside a Plexiglas tube with a variable head. The data thus obtained were utilized to generate a plot that depicted the cumulative water volume as a function of time. This graphical representation facilitated the determination of the steady-state flow rate. The permeability test was conducted using a flexible-wall permeability tester (Nanjing Geotechnical Instrument & Equipment Co., Ltd., Nanjing, China) in accordance with ASTM D5084-16 [33]. Saturated specimens were tested under controlled hydraulic gradients to ensure steady-state flow conditions, with permeability coefficients calculated using Darcy’s law, as illustrated in Equation (3).

(3) $$K_{v}=2.3\;aL/At\cdot lg\;(H_{1}/H_{2})$$

where *K_v_* is the water permeability coefficient, *a* is the cross-sectional area of the glass tube, *A* is the specimen area, *t* is the penetration time, *L* is the length of the specimen, *H*_1_ is the initial head height, and *H*_2_ is the height of the descending head.

#### 2.3.5. Nuclear Magnetic Resonance Test

Nuclear Magnetic Resonance (NMR) is a non-destructive analytical technique based on the interaction of hydrogen nuclei with external magnetic fields. When a sample is placed in a static magnetic field, hydrogen nuclei in water molecules align with the field. A radiofrequency pulse perturbs this alignment, and the subsequent relaxation of the nuclei generates detectable signals. Through the meticulous analysis of the intensity and spatial distribution of NMR signals, researchers are able to glean valuable insights into the alterations occurring in the pore structure distribution within the specimen under study. The focus is on exploring the microscopic mechanisms responsible for the changes in pore structure as a result of the variation in rubber particle size and content. This technique provided direct evidence of how rubber particle size and content influence pore structure, a key factor controlling mechanical performance. The analysis is carried out by employing a MicroM12-025VR NMR core analyzer purchased from Shanghai Electronic Technology Co., Ltd. (Shanghai, China). The samples had a diameter of 18 mm and a height of 30 mm. Before the commencement of the testing process, the samples were saturated under back pressure, and any surface water present on them was carefully removed. Employing the MicroM12-025VR NMR core analyzer, the T2 decay maps of pure water within the samples were measured. During the entire experiment, the temperature was strictly maintained at 32 °C.

## 3. Results and Discussion

### 3.1. Effect of Rubber Particle Sizes and Content on Calcium Carbonate Content

**Figure 5:**
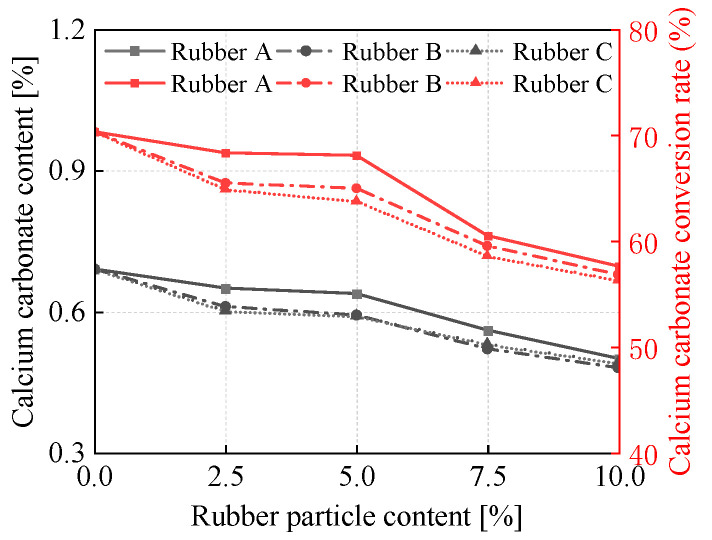
Variations of CaCO_3_ content and conversion rate for the testing samples with different rubber particle sizes and contents.

As illustrated in Figure 5 and Table 4, the calcium carbonate content in the samples treated by EICP at various rubber particle sizes and contents was analyzed. It can be observed that as the rubber particle content rises, both the calcium carbonate content and the conversion rate of the specimens decline. In general, when the rubber particle content is fixed, an increase in particle size leads to a reduction in both the calcium carbonate content and the conversion rate. Moreover, there is an increasing disparity in calcium carbonate content among the three specimens treated with rubber particles when higher rubber particle contents are employed. Notably, the specimen incorporating rubber A exhibits higher calcium carbonate content and conversion rate compared to others. The samples with smaller amounts of rubber particles can maintain a high level of calcium carbonate conversion across all three particle sizes. This suggests that a higher content of rubber particles negatively impacts calcium carbonate precipitation due to its hydrophobic nature [24]. Additionally, the absence of intra-granular pores in larger-sized rubber particles may account for lower overall levels of calcium carbonate content and conversion rate observed for Rubber C relative to the other two sizes [18].

### 3.2. Triaxial Compression Test

#### 3.2.1. Stress–Strain Relationship

**Figure 6:**
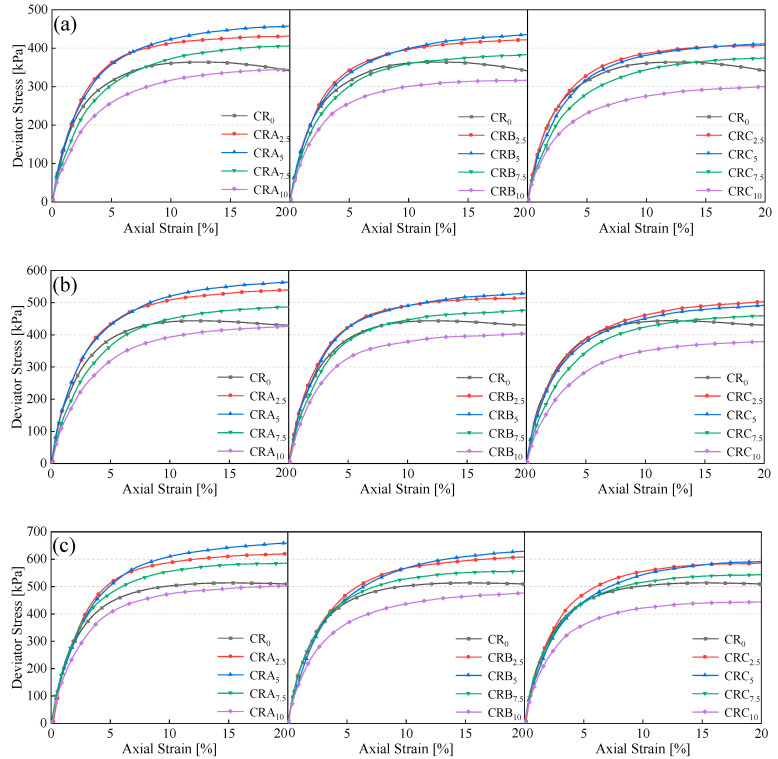
Stress–strain curves of EICP-RC solidified clay under different confining pressure: (**a**) 50 kPa, (**b**) 100 kPa, (**c**) 150 kPa.

Figure 6 depicts the typical stress–axial strain relationship curves for the EICP-treated samples under confining pressures of 50, 100, and 150 kPa. As is evident from the figure, the peak stress of the EICP-treated specimens shows an upward trend with the increase in confinement pressure. Once the peak stress is attained, the stress diminishes as the axial strain grows. Moreover, in the triaxial test, the specimens treated solely with EICP (CR_0_) display strain-softening behavior.

As shown in Figure 6, the EICP-treated clay (CR_0_) exhibits a distinct strain-softening behavior with a clear stress peak, characteristic of brittle failure. In contrast, specimens incorporating rubber particles (CRA, CRB, CRC) display strain-hardening behavior without observable peak stresses. This transition is attributed to the elastomeric properties of rubber, which dominate the post-yield response by dissipating energy through reversible deformation and interfacial debonding. Notably, the absence of peaks in rubber-modified specimens aligns with their ductile failure mechanism, as reported in rubber-reinforced soils [34,35]. Moreover, the inclusion of rubber particles results in enhanced strength, suggesting that the elastic reaction of rubber particles during the compression process contributes to the prevention of cracking [36]. Furthermore, EICP has a reinforcing effect between rubber particles and clay particles [37]. As is observable from Figure 6a, the specimens incorporating small-sized rubber particles exhibit higher strength compared to those with large-sized rubber particles. This phenomenon can be ascribed to the fact that the addition of small-sized rubber powder expands the area of the relatively flat surface of the soil. Simultaneously, it decreases the volume of pores and smoothens the surface [25]. Effective calcium carbonate refers to the calcium carbonate that plays a role in bonding adjacent soil particles [38], and smooth surfaces are more conducive to the production of effective calcium carbonate, increasing the existing bonds between particles [39]. As the size of the rubber particles increases, the peak stress shows a decreasing trend. This could potentially be attributed to the transformation of the rubber particles’ function. Initially, they fill the pores, but as their size grows, they gradually become part of the soil skeleton. This transition leads to a reduction in the bearing capacity of the soil skeleton and the formation of relatively large pores within the structure.

It can be observed that as the confining pressure rises, the stress peak is postponed and gradually fades away. There are reasons for this phenomenon: (1) Pore instability increases with increasing particle contact stress [40]; (2) under localized high-stress conditions, the particles are in closer contact with each other, resulting in an augmentation of the coefficient of friction among the particles [41]. Compared with the low confining pressure condition (Figure 6a), the specimens with different rubber particle contents under the high confining pressure condition have higher peak stresses (Figure 6c), suggesting that the influence of rubber particle content is associated with the external load.

#### 3.2.2. Cohesion and Internal Friction Angle

In accordance with the Mohr–Coulomb yield criterion, the internal friction angle and cohesion at the peak state of EICP-RC solidified clay were ascertained for various rubber particle sizes and content levels.

**Figure 7:**
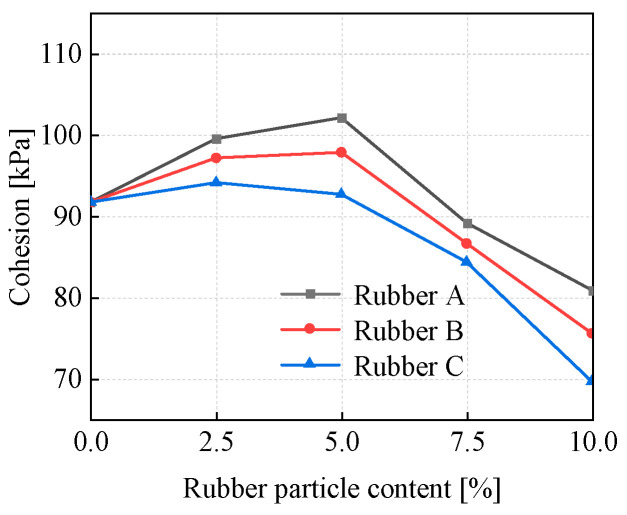
The cohesion of specimens in peak state.

As presented in Figure 7, which takes into account three particle sizes (Rubber A, Rubber B, and Rubber C). The addition of rubber particles significantly increased the cohesion of the samples compared to the samples without rubber particles. With the increase in rubber particle content, the cohesive force of the specimens first exhibits an upward trend and subsequently a downward trend. As the rubber particle content increases, the cohesive force of the specimens initially shows an increasing tendency and then a decreasing one. In the case of the specimens with Rubber C added, the cohesion reaches its peak value when the rubber particle content is 2.5%. For the specimens with Rubber A and Rubber B incorporated, the cohesion reaches its maximum when the rubber particle content is 5%. The incorporation of an appropriate amount of rubber particles exerts a positive influence on the cohesion of the specimens. This can be ascribed to the fact that the addition of rubber particles leads to a reduction in the porosity of the specimens and suppresses the development of cracks [35]. The rubber particles exhibit greater elasticity and a more pronounced small-amplitude rebound effect during testing, leading to higher peak stresses within the specimen [42]. Simultaneously, EICP caused the formation of CaCO_3_ crystals between the soils. This process established structural continuity and enhanced the bonding strength between soil particles and rubber particles [43]. When the content of rubber particles exceeded 5%, the cohesion of the specimens was generally lower than that of samples without added rubber particles. The findings indicated that the cohesion of the mixed soil mass was influenced by the combined impact of EICP and rubber particles. The distribution patterns and interactions of rubber particles of different sizes within the clay exerted a substantial influence on the cohesion of the mixed soils. Smaller-sized rubber particles have a greater propensity to fill the pores between clay particles. This filling action increases the contact points and friction among the particles. Moreover, it promotes the production of effective CaCO_3_ in the EICP process, thereby enhancing the cohesion of the soil mixture. Conversely, when the size of the rubber particles is excessively large, the soil forms larger agglomerates. As a result, the effective contact area between the rubber particles and the clay particles is reduced, and the amount of effective CaCO_3_ produced by EICP is diminished, ultimately resulting in a decrease in cohesion. With the decrease in rubber particle size, the point at which peak cohesion occurs gradually shifted towards higher rubber particle contents. The outcomes demonstrated that the augmentation of cohesion was mainly dependent on the strengthening effect brought about by EICP, along with the size and content characteristics of the rubber particles.

**Figure 8:**
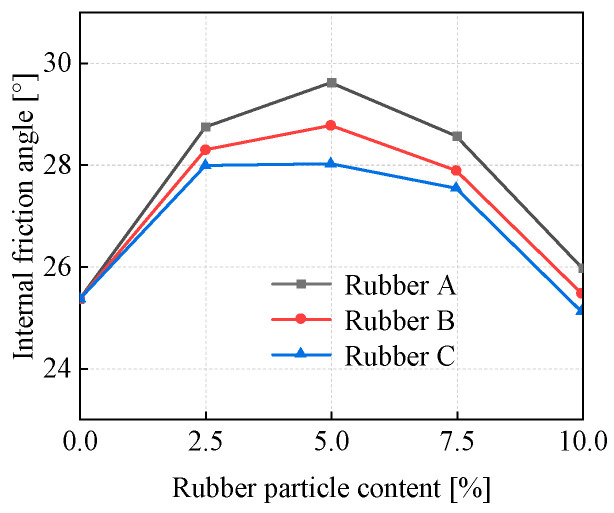
The internal friction angle of specimens in peak state.

Figure 8 shows the influence exerted by the size and content of rubber particles on the internal friction angle within EICP-RC solidified clay. The trend in internal friction angle mirrors that of cohesion. Three particle sizes (Rubber A, Rubber B, and Rubber C) were examined. As the rubber particle content increases, the internal friction angle of the specimens first exhibits an upward trend and then a downward one. For all three particle sizes, it reaches its peak value when the rubber particle content is 5%. In addition, for the EICP-treated specimens, the internal friction angle increases as the size of the rubber particles decreases. This phenomenon can be ascribed to two primary factors: (1) The filling effect of rubber particles, which occupy soil pores, reduces porosity, and enhances soil compactness. As rubber particle content increases and size decreases, this filling effect becomes more pronounced, further improving soil compactness and increasing the internal friction angle [44]. The results of NMR tests also proved the reduction of porosity. (2) The frictional interaction at the interface between rubber particles and soil, which also boosts the internal friction angle. Smaller rubber particles lead to a larger contact area with the soil, thereby increasing interfacial friction and further elevating the internal friction angle [45].

### 3.3. Oedometer Test

#### 3.3.1. Stress–Strain Relationship

**Figure 9:**
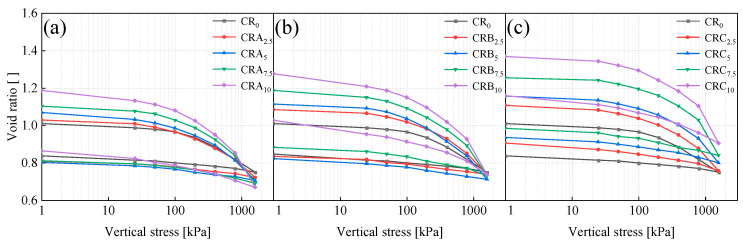
The void ratio–vertical stress relationship curves of samples treated with EICP under different particle sizes (**a**) Rubber A, (**b**) Rubber B, and (**c**) Rubber C.

During the loading and unloading processes, the deformation of three specimens featuring different particle sizes and four distinct rubber particle contents was measured. This measured deformation was then utilized to estimate the pore ratio. Figure 9 depicts the variation of the pore ratio with vertical stress for all the specimens. As illustrated in Figure 9, the pore ratio exhibits a decreasing trend with the increase in vertical stress. For all specimens during the loading path, the most significant change in the pore ratio occurs when the vertical stress rises from 800 kPa to 1600 kPa. In contrast to the loading path, during the unloading path, as the vertical stress decreases from 1600 kPa to 0 kPa, the vertical strain increases in an almost linear fashion. With the increase in rubber particle content, both the change in the pore ratio during each loading step and the difference in the pore ratio between successive loading steps become more pronounced. Additionally, it can be observed that the initial pore ratio of the specimens rises as the rubber particle content increases. During the subsequent unloading steps, the pore ratios of all specimens increase in a similar manner as the vertical stress decreases.

Comparing Figure 9a–c, it is evident that the pore ratios of the specimens increase as the particle size of the rubber particles increases. This finding indicates that the rubber particle size has a significant impact on the porosity or sparseness of the soil structure. Moreover, it was noted that, under a vertical stress of 1600 kPa, specimens containing smaller rubber particles had lower pore ratios compared to specimens without rubber particles. A reduced pore ratio signifies tighter soil particle contact and greater soil density. This further illustrates that smaller rubber particles primarily achieve their filling effect through interparticle stacking, effectively occupying soil pores due to their compact arrangement, thereby enhancing soil denseness. Conversely, larger rubber particles predominantly fill the soil through bridging, resulting in a looser particle combination and generally higher pore ratios.

#### 3.3.2. Compressibility

**Figure 10:**
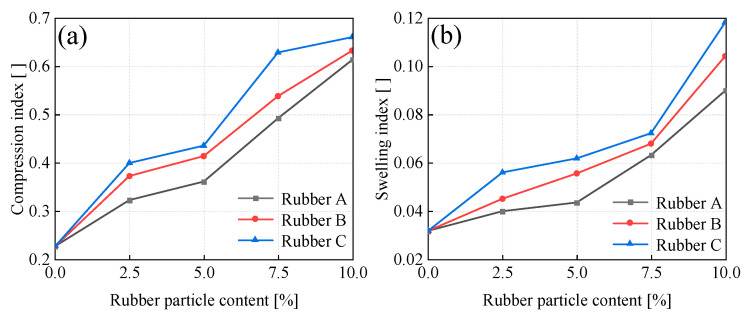
(**a**) Variation of compression indices (Cc) with rubber content; (**b**) Variation of swelling indices (Cs) with rubber content.

To comprehensively characterize the compressibility and elastic recovery behaviors of rubber-reinforced soils, the compression index (C_c_) and swelling index (C_s_) were systematically quantified through consolidation testing. The compression index (C_c_) was obtained from the slope of the deformation curve within the vertical stress range of 800–1600 kPa, while the swelling index (C_s_) was determined from the slope of the deformation curve in the vertical stress range of 1600–800 kPa. C_c_ is used to predict long-term settlement in foundation design, while C_s_ is used to quantify recoverable deformation, which is essential for assessing structural resilience under cyclic loading conditions. As depicted in Figure 10, the values of C_c_ and C_s_ were graphed in relation to the rubber particle content. With the increase in rubber particle content, both indices display an upward-trending behavior. The compressibility of soil particles is influenced by a multitude of parameters, such as the initial pore ratio, the shape of the particles, the mineralogical composition, and particle size distribution [46]. Among these factors, the initial pore ratio has been determined to be the most crucial element influencing the compressibility of soil particles [47]. As illustrated in Figure 9, as the rubber particle content increases, the quantity of porous rubber particle aggregates also increases, resulting in an augmented initial pore ratio in the specimen. Additionally, during the loading process, the deformation of specimens with a high rubber particle content and a high initial pore ratio becomes more substantial. As a consequence, the value of C_c_ increases in tandem with the growth of the rubber particle content within the specimen.

During unloading, a portion of the deformation that the samples underwent during loading is reversible. This phenomenon can be ascribed to the release of the elastic energy that was stored within each soil particle during the loading phase [48]. Furthermore, the elastic deformation of the soil is subject to the influence of particle stiffness [49]. Smaller rubber particles are more effective at filling the pores, and their higher specific surface area results in increased contact and interaction among particles, thereby enhancing the specimen’s stiffness. Numerous studies have indicated that with the increase in the stiffness of sample particles, the elastic deformation during unloading is diminished [50,51]. As the particle size of rubber particles increases, their elastic characteristics may become more significant, contributing to an enhancement in the resilience index of the soil.

### 3.4. Effect of Rubber Particle Size and Content on Permeability

**Figure 11:**
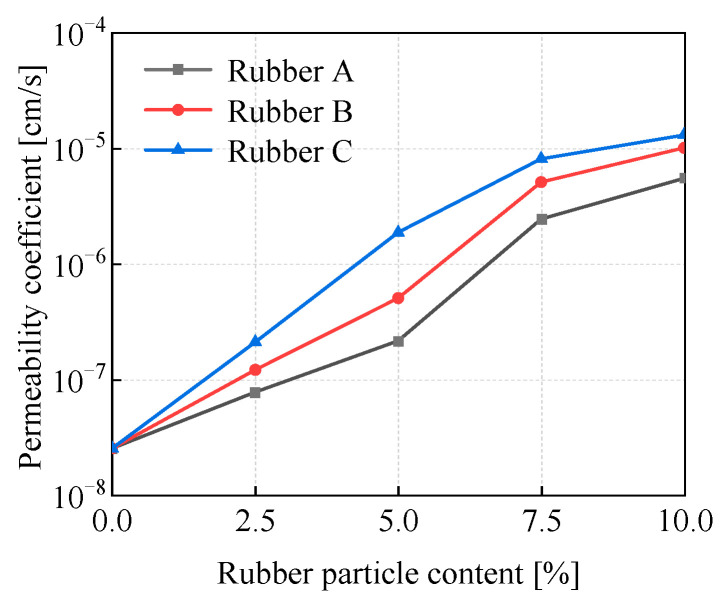
Variation in the permeability of samples with rubber particle content.

Figure 11 depicts how the content and size of rubber particles exert an influence on the permeability coefficient of the EICP-RC solidified clay. The permeability coefficients show a consistent increase with the rising rubber particle content, peaking at a maximum value at 10% rubber particle content. Clearly, for all samples, the permeability coefficients generally show an upward trend with the increase in rubber particle content. Notably, a substantial increase is witnessed when the content surpasses 7.5%. At 10% rubber particle content, the permeability coefficients of rubber A, rubber B, and rubber C modified clay samples reach 5.54 × 10^−6^, 1.01 × 10^−5^, and 1.31 × 10^−5^ cm/s, respectively. The increase in permeability coefficients of the samples modified using larger-sized rubber particles can be ascribed to the larger continuous surface area. This larger surface area promotes the movement of water within the modified clay samples [52]. Figure 11 demonstrates that for specimens with diverse rubber particle sizes, the permeability coefficients display a comparable upward trend as the rubber particle content grows. Nevertheless, the degree of the increase in the permeability coefficient is contingent upon the size of the rubber particles. In the case of the modified clay samples incorporating Rubber C, the permeability coefficient experiences an almost linear growth, and this growth plateaus as the rubber particle content continues to increase. In the modified clay samples containing Rubber A and Rubber B, when the rubber particle content is below 5%, the permeability coefficients rise in a gradual manner. However, once the content reaches 7.5%, there is a sharp escalation. The change in the permeability coefficient is affected by multiple factors: the interfacial characteristics of the rubber–soil particles, the connectivity within the pore structure, and the tortuosity of the flow paths. The introduction of rubber particles not only enlarges the pore volume in the soil but also initiates water flow at the interface between the clay particle matrix and the rubber particles, thereby resulting in an overall enhancement of the permeability coefficient. Additionally, the inclusion of smaller rubber particles causes an increase in the tortuosity of the water migration paths [53]. This explains why the permeability coefficients of rubber A-modified samples are generally lower than those of rubber B and rubber C.

### 3.5. Nuclear Magnetic Resonance Analysis

**Figure 12:**
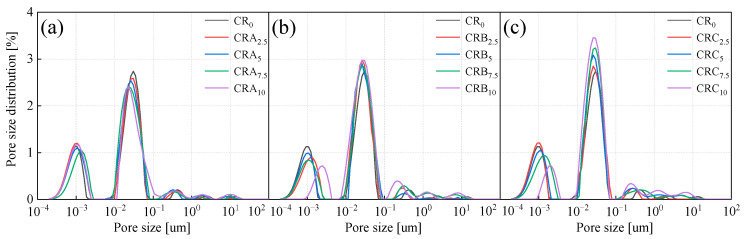
Pore size distribution under different rubber sizes and contents (**a**) Rubber A, (**b**) Rubber B, and (**c**) Rubber C.

The pore structure exerts a direct influence on the strength of EICP-RC solidified clay. An excessive amount of pore space or non-uniform pore distribution can lead to a reduction in soil density, and in turn, have an impact on its mechanical properties. As a result, a quantitative analysis of the pore interiors within the specimens is of great necessity. Nuclear magnetic resonance (NMR) testing represents a vital approach for characterizing the internal porosity of soil. By means of the NMR curve, the number and distribution of pores in the sample can be directly visualized. Figure 12 depicts the influence exerted by different rubber particle sizes and contents on the porosity of EICP-RC solidified clay. The number of pores is determined by calculating the area enclosed by the NMR curves. In accordance with the NMR curve and its three characteristic peak areas, pores are classified into micropores (with a diameter of less than 2 µm), mesopores (ranging from 0.2 to 7 µm), and macropores (with a diameter greater than 7 µm). The NMR curve is predominantly characterized by the first two peaks, indicating that most of the pores in the specimen are small and mesopores. As Figure 12 demonstrates, as the rubber particle content rises, the rightward displacement of the characteristic peaks denotes an augmentation in pore size. Simultaneously, the upward trend of these peaks implies an increase in the quantity of pores. Therefore, the enlargement of pore size and the increase in pore number are identified as the primary reasons for the decline in mechanical properties due to higher rubber particle content.

Comparing Figure 12a–c, in the situation of adding Rubber A, with the increase in rubber particle content, the characteristic peaks corresponding to medium-sized and small pores gradually decline. Conversely, the characteristic peaks associated with large pores continuously increase. This suggests that smaller rubber particles are more proficient in filling the pores within the specimens, thereby leading to a reduction in the number of large pores. In the case of adding Rubber B, as the rubber particle content escalates, the characteristic peaks of small pores exhibit a rightward shift, which implies an expansion in pore size. The medium pore characteristic peaks remain relatively stable, but the large pore characteristic peaks gradually increase, a trend consistent with samples containing rubber C. The pore size corresponding to the small pore characteristic peaks also increases with rising rubber particle content. This evidence shows that with the increase in rubber particle size, the pores inside the sample enlarge, consequently resulting in a decline in mechanical properties. This finding is consistent with the outcomes of the triaxial test.

**Figure 13:**
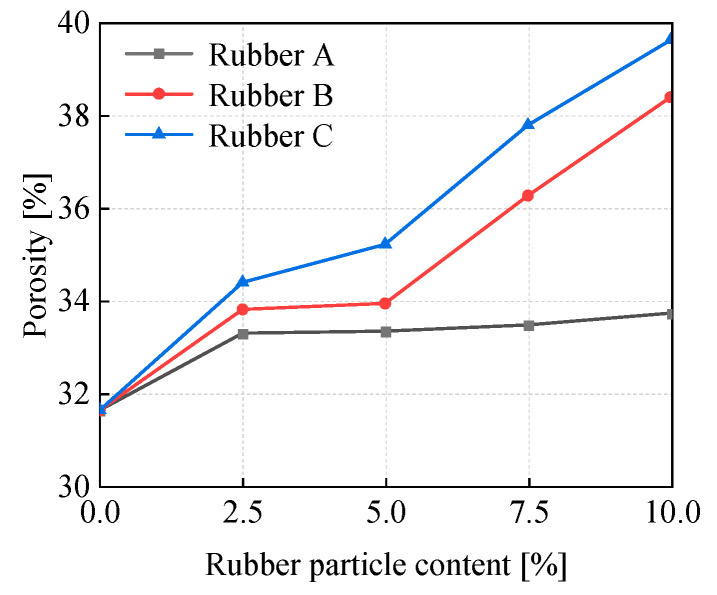
Porosity of samples at different rubber particle sizes and contents.

Figure 13 compares the porosity of samples with different rubber particle sizes under varying content levels, illustrating the dynamic evolution of solidified soils with changes in rubber particle content for each type. For rubber particle content ranging from 0 to 5%, the porosity increases slightly, reaching only 1.71% to 3.59%. However, when the content ranges from 5% to 10%, the porosity increases significantly, reaching up to 4.46%. As the rubber particle content remains constant, the porosity of the specimens shows a gradual upward trend as the rubber particle size increases. This implies that larger-sized rubber particles play a role in enhancing the overall porosity within the specimens.

### 3.6. Microstructure Change Process

**Figure 14:**
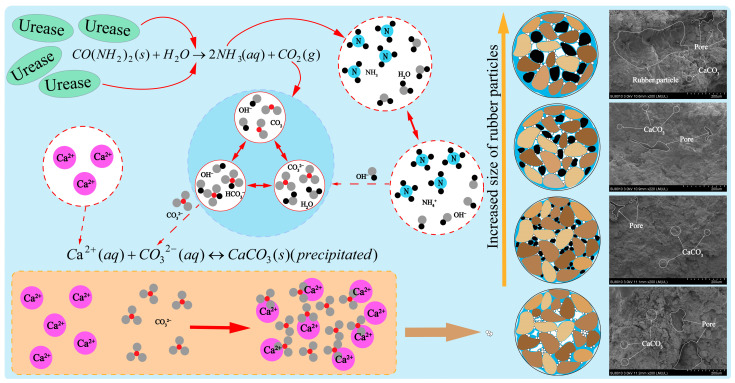
Evolution process of the microstructure of EICP-RC solidified clay.

The microstructure and mechanical tests of the EICP-RC solidified clay elucidated the primary mechanisms governing pore structure changes during its evolution (Figure 14). The strength of the solidified clay is mainly determined by two key factors: the calcium carbonate content and the interfacial adhesion between rubber particles and clay particles. During the early phase of the reaction, urease acts on urea through hydrolysis, generating ammonia and carbon dioxide. This chemical process leads to the establishment of an alkaline environment, which in turn promotes the formation of carbonate ions. In the subsequent stages, dissolved calcium ions are adsorbed onto the surfaces of either soil particles or rubber granules. This adsorption process triggers the precipitation of amorphous or low-crystallinity calcium carbonate. Subsequently, these reaction products enclose the soil particles and fill the pores, ultimately giving rise to a dense, monolithic structure.

It was noted that with the increase in the particle size of rubber particles, the pore structure of the solidified soil experienced substantial alterations. The addition of smaller rubber particles not only efficiently filled the pores but also offered a larger specific surface area. As a result, the contact area for the precipitation of calcium carbonate was augmented, leading to an increase in the effective calcium carbonate content. These calcium carbonate crystals interlock to form a supporting framework, which is conducive to the enhancement of mechanical properties [38]. As the particle size of the rubber particles increases, they gradually become incorporated into the soil skeleton. This integration process gives rise to the formation of larger pores within the soil matrix. The difference in stiffness between the rubber particles and the soil particles leads to a weakening of the overall load-bearing capacity of the soil structure. Additionally, the hydrophobic nature of the larger continuous surfaces of bigger rubber particles results in increased ineffective calcium carbonate formation. In addition, the elastic characteristics and the relatively low elastic modulus of rubber particles lead to a decline in the interlocking efficacy among adjacent particles [53].

## 4. Conclusions

In this experimental investigation, the focus is on exploring the potential of applying EICP as a consolidation technique and introducing rubber particles, which account for 2.5–10% of the dry mass, as an additive to enhance the geotechnical properties of weak clays. Three sizes of rubber particles were systematically examined. The research aims to comprehensively evaluate the strength, consolidation behavior, swelling characteristics, and permeability of the EICP-treated clay that has been solidified with rubber particles. The following conclusions can be drawn from the test results:

(1) The addition of rubber particles exerts a certain influence on the degree of the EICP reaction. As the rubber particle content rises, the calcium carbonate content in the samples generally shows a downward trend. Nevertheless, the inclusion of rubber particles is capable of enhancing the shear strength of the specimens. Specifically, both the cohesion and the angle of internal friction of the EICP-RC solidified clay increase as the rubber particle content increases. For a specific rubber particle size, the most significant improvement occurs when the rubber particle content reaches 5%. Moreover, for any fixed rubber particle content, smaller-sized rubber particles demonstrate a more pronounced improvement effect.
(2) According to the findings of the odometer test, as the rubber particle content in the specimen augmented, its deformation also increased, accompanied by the growth of both the C_c_ and C_s_. When the rubber particle content remained constant, the samples treated with large-sized rubber particles exhibited a larger Cc and Cs. The results of the permeability test indicated that the permeability coefficient of the samples rose with the increase in rubber particle content. The size of the rubber particles had a notable impact on the permeability of the samples. Generally, the permeability coefficients of the samples modified with large-sized rubber particles were higher than those of the samples modified with small-sized rubber particles.
(3) Incorporating large-sized rubber particles into cemented soils modifies the soil’s pore structure by increasing both the size and number of mesopores and macropores. Conversely, adding small-sized rubber particles has minimal impact on overall pore structure but effectively fills mesopores and macropores. The synergistic effect between EICP and rubber particles can more effectively solidify the soil structure.

In this study, macroscopic outcomes are integrated with microscopic analyses to lucidly illustrate the influence of particle size and content on the cementation effect, as well as the synergistic effect between EICP and rubber particles. This research indicates that the employment of rubber particles to enhance the geotechnical properties of EICP-treated clay may confer multiple advantages. The utilization of rubber particles for clay improvement not only results in the consumption of a substantial number of used rubber tires but also enhances the geotechnical properties of the clay. While this study demonstrates the effectiveness of EICP-RC composites in enhancing clay’s mechanical properties, key limitations include the laboratory-scale focus (lacking field validation), unquantified long-term durability under environmental cycles (wet–dry, freeze–thaw), and potential ecological risks from urea byproducts. The rubber particles, though repurposed from waste tires, may raise concerns about heavy metal leaching or microplastic generation over time. Future research should prioritize field trials to assess scalability, investigate rubber aging resistance, and evaluate long-term environmental impacts to ensure sustainable application.

## Figures and Tables

**Table 1:** Index properties of the soil.

Liquid Limit (%)	Plastic Limit (%)	Plasticity Index (%)	Specific Gravity
42.80	24.06	18.74	2.69

**Table 2:** XRF testing result for the soil.

Component	SiO_2_	Al_2_O_3_	CaO	Fe_2_O_3_	MgO	K_2_O	TiO_2_	Na_2_O	P_2_O_5_	SrO	Rb_2_O
Mass (%)	52.96	18.75	11.80	7.36	3.86	3.42	0.79	0.40	0.21	0.05	0.01

**Table 3:** Compaction test results.

Group	Rubber Content (%)	Optimum Moisture Content (%)	Maximum Dry Density (g/cm^3^)
CR_0_	0	24.42	1.59
CRA_2.5_	2.5	23.45	1.55
CRA_5_	5	23.06	1.51
CRA_7.5_	7.5	22.72	1.5
CRA_10_	10	21.00	1.48
CRB_2.5_	2.5	22.90	1.56
CRB_5_	5	22.28	1.48
CRB_7.5_	7.5	21.17	1.45
CRB_10_	10	20.33	1.42
CRC_2.5_	2.5	22.74	1.55
CRC_5_	5	22.71	1.55
CRC_7.5_	7.5	22.09	1.53
CRC_10_	10	21.09	1.49

**Table 4:** Acid washing experiment results.

Group	Rubber Content (%)	CaCO3 Content (%)	CaCO3 Conversion Rate (%)
CR_0_	0	0.69	70.31
CRA_2.5_	2.5	0.649	68.33
CRA_5_	5	0.638	68.09
CRA_7.5_	7.5	0.56	60.49
CRA_10_	10	0.5	57.61
CRB_2.5_	2.5	0.61	65.47
CRB_5_	5	0.592	64.98
CRB_7.5_	7.5	0.52	59.52
CRB_10_	10	0.48	56.82
CRC_2.5_	2.5	0.601	64.87
CRC_5_	5	0.59	63.75
CRC_7.5_	7.5	0.53	58.58
CRC_10_	10	0.49	56.26

## Data Availability

The original contributions presented in this study are included in the article. Further inquiries can be directed to the corresponding authors.
